# REPP: A robust cross-platform solution for online sensorimotor synchronization experiments

**DOI:** 10.3758/s13428-021-01722-2

**Published:** 2022-02-11

**Authors:** Manuel Anglada-Tort, Peter M. C. Harrison, Nori Jacoby

**Affiliations:** 1 Computational Auditory Perception Group, Max Planck Institute for Empirical Aesthetics, Frankfurt am Main, Germany; 2 Centre for Music and Science, Faculty of Music, University of Cambridge, Cambridge, UK

**Keywords:** Sensorimotor synchronization, Rhythm, Movement, Timing, Online experiments

## Abstract

Sensorimotor synchronization (SMS), the rhythmic coordination of perception and action, is a fundamental human skill that supports many behaviors, including music and dance (Repp, 2005; Repp & Su, 2013). Traditionally, SMS experiments have been performed in the laboratory using finger tapping paradigms, and have required equipment with high temporal fidelity to capture the asynchronies between the time of the tap and the corresponding cue event. Thus, SMS is particularly challenging to study with online research, where variability in participants’ hardware and software can introduce uncontrolled latency and jitter into recordings. Here we present REPP (Rhythm ExPeriment Platform), a novel technology for measuring SMS in online experiments that can work efficiently using the built-in microphone and speakers of standard laptop computers. In a series of calibration and behavioral experiments, we demonstrate that REPP achieves high temporal accuracy (latency and jitter within 2 ms on average), high test-retest reliability both in the laboratory (*r* = .87) and online (*r* = .80), and high concurrent validity (*r* = .94). We also show that REPP is fully automated and customizable, enabling researchers to monitor experiments in real time and to implement a wide variety of SMS paradigms. We discuss online methods for ensuring high recruiting efficiency and data quality, including pre-screening tests and automatic procedures for quality monitoring. REPP can therefore open new avenues for research on SMS that would be nearly impossible in the laboratory, reducing experimental costs while massively increasing the reach, scalability, and speed of data collection.

**Supplementary Information:**

The online version contains supplementary material available at 10.3758/s13428-021-01722-2.

Sensorimotor synchronization (SMS) is a fundamental human skill that involves the temporal coordination of rhythmic movement with a predictable external event (Repp, 2005; Repp & Su, 2013). SMS requires individuals to precisely integrate visual or auditory perception with motor production, supporting a wide range of human behaviors. For example, the ability to entrain to an external auditory cue plays a key role in musical experiences across human cultures (Savage et al., 2015; Jacoby et al., 2021) and has been linked to specific genotypes, suggesting an innate human sensitivity to rhythm (Niarchou et al., 2021). SMS has also been associated with the development of literacy skills, such as reading and speech (Carr et al., 2014; Flaugnacco et al., 2014; Ladányi et al., 2020; Tierney & Kraus, 2013), and various neurodevelopmental disorders, including attention deficit hyperactivity disorder (Noreika et al., 2013) and Parkinson’s disease (Bieńkiewicz & Craig, 2015).

Quantitative research on SMS dates back at least to 1886 (Stevens, 1886), but its popularity has increased considerably in recent decades (see Repp, 2005; Repp & Su, 2013, for reviews). SMS experiments can differ substantially in their implementation, using different production modes (e.g., finger tapping, clapping, or speaking), different stimulus domains (e.g., visual or auditory), and different experimental designs, including rate limits (London, 2002), perturbation studies (Repp, 2002a), simulated partners (Repp & Keller, 2008), and transmission chains (Jacoby & McDermott, 2017; Ravignani et al., 2016). However, at their core, most SMS experiments consist of a relatively simple procedure: participants tap with their index finger to a rhythmic sequence of auditory stimuli. This procedure presents a methodological challenge: how to measure the asynchrony (or synchronization error) between the time of the tap and the corresponding cue event with high millisecond-level precision. To meet this challenge, previous studies have used various laboratory-based methods that rely on specialized software and hardware. For example, some studies have used external hardware to record responses and control auditory feedback (e.g., a MIDI percussion pad or keyboard connected to computer software), such as *FTAP* (Finney, 2001) and *Max-MSP* (Patel et al., 2005; Repp et al., 2005). Researchers have also proposed solutions that use the low-level timing hardware of Arduino microcontrollers, including *TapArduino* (Schultz & van Vugt, 2016) and *TeensyTap* (van Vugt, 2020). Another popular solution is *MatTAP*, a MATLAB-based toolbox for dedicated data acquisition hardware (Elliot et al., 2009). Others have developed an iOS application for tapping experiments that takes advantage of specific hardware in mobile Apple devices (*Tap-It*, Kim et al., 2012). In previous work, we have used a simple, low cost, in-lab method that achieves high temporal fidelity by simultaneously recording the audio stimulus and tapping responses using a standard sound card with an audio loopback cable (Elliott et al., 2018; Jacoby & McDermott, 2017; see Experiment 2 for a description of this method).

Nevertheless, none of these methods are viable for performing SMS experiments over the internet, where researchers have very limited control over participants’ hardware and software. This lack of experimental control combined with the technical demands of SMS tasks makes studying SMS with online research a true challenge. In particular, SMS experiments performed online, such as tapping on the spacebar or mouse in synchronization to an external beat, can introduce all kinds of delay in latency and jitter into the recorded timestamps (Anwyl-Irvine et al., 2020; Bridges et al., 2020). Latency refers both to the time gap between a participant pressing a key and the device registering the keypress, and the time interval between initiation of audio playback and the physical start of the sound. It is often related to issues concerning scan rates, device drivers, internet connection, operating system variability, and sound card start-up latencies. Jitter is closely related and refers to the variation in latency. It can be either introduced in each tapping onset or across tapping trials (e.g., operating systems usually process each keyboard stroke with different temporal latencies). These inaccuracies can be in the order of 60 to 100 ms and can vary considerably between platforms, browsers, and devices (Anwyl-Irvine et al., 2020). Thus, measuring participants' asynchronies in online settings with high precision is currently unfeasible.

Another important source of noise in online experiments is altered participant behavior compared to laboratory settings (e.g., Clifford & Jerit, 2014). This can be challenging in SMS tasks because they usually require participants to pay close attention to the task and take a large number of trials per session. There is also a higher risk of fraudulent responders (Ahler et al., 2019; Crump et al., 2013), including both computer “bots” and non-serious respondents, such as participants who do not tap at all or tap at a regular rate irrespective of the external auditory cue. When performing online research on SMS, it is therefore important to analyze experimental trials and monitor performance in real time, providing feedback to participants and excluding fraudulent responders.

Here we present REPP (Rhythm ExPeriment Platform), a novel technology for measuring SMS in online experiments that can work efficiently using hardware and software available to most participants online, specifically standard laptops with working speakers and microphones. To address core issues related to latency and jitter, REPP uses a free-field recording approach: the audio stimulus is played through the laptop speakers and the original signal is simultaneously recorded with participants’ tapping response using the built-in microphone (Fig. 1a). The success of this method relies on a simple observation: although the initial onset in a recording is hard to control due to the interplay between the sound card and operating system, once a sound card starts recording, it registers all subsequent sound events as audio samples encoded with high precision with respect to the beginning of the recording. Thus, by using a single audio recording to simultaneously capture the stimulus and tapping onsets, we can remove the most significant sources of delay in both response and presentation latencies. We then apply audio filtering and other signal processing techniques to the resulting audio recording to split the different components of the recording into separate channels and therefore extract the stimulus and tapping onsets with reliable timing. Finally, we use custom markers with known temporal locations to unambiguously identify the position of the tapping and stimulus onsets in the audio recording, allowing a precise alignment to measure participants' asynchronies. REPP can be executed rapidly in real time and is fully customizable, enabling researchers to adapt the code to support a wide range of SMS paradigms in online and laboratory settings. In this paper, we aim to validate REPP in a series of experiments while demonstrating how to best implement it in online studies to ensure high data quality.

**Fig. 1:**
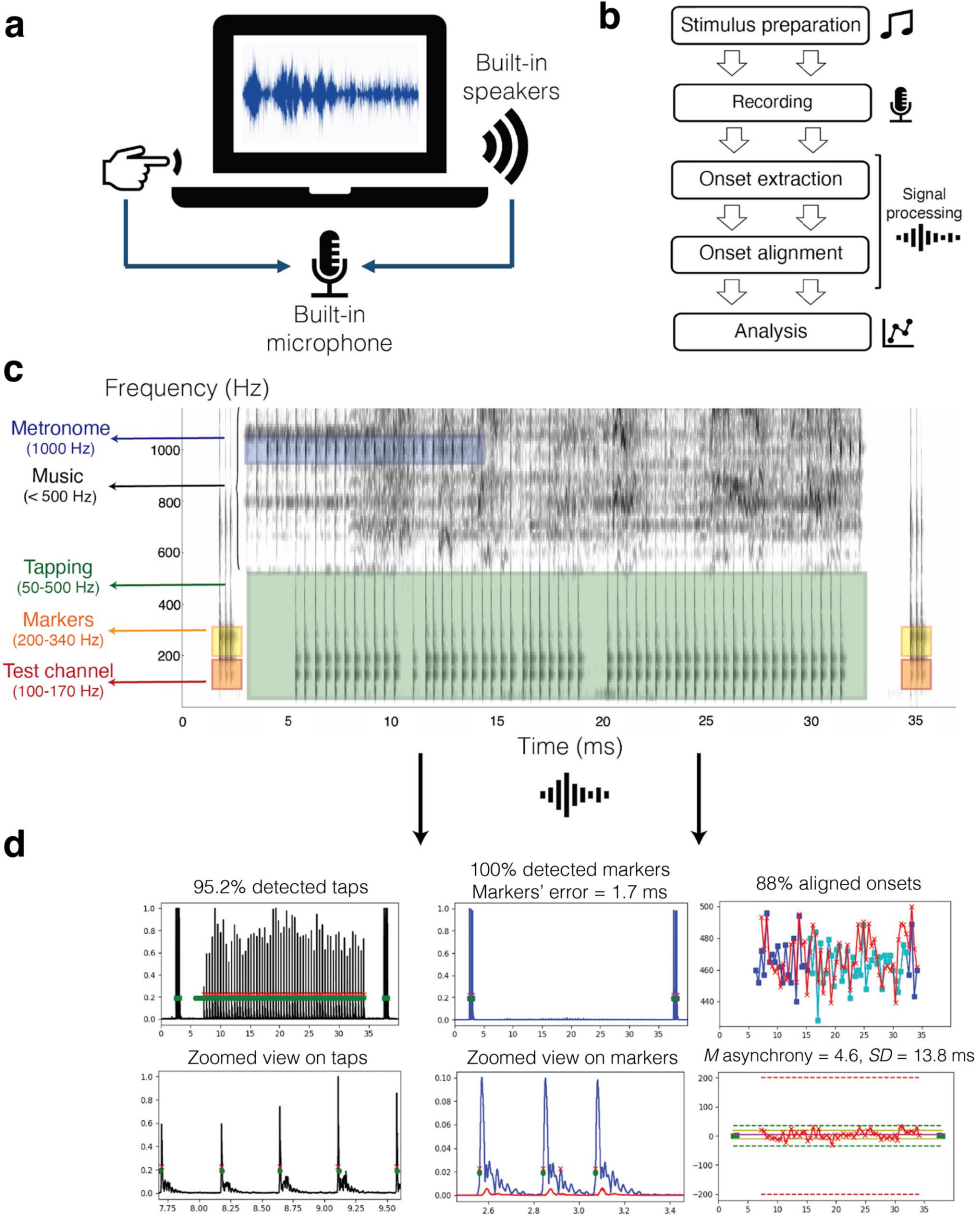
REPP: A robust cross-platform solution for online SMS experiments. **a** REPP uses a free-field recording approach that can work efficiently using standard hardware and software available to most online participants. **b** REPP comprises five main steps. **c** Example of a recording using REPP in a trial of beat synchronization to music. REPP uses a unique frequency range for each audio element in the recording: metronome (blue), tapping (green), markers (yellow), and test channel (red). **d** Output of the performance analysis after the signal processing steps, including the number of detected tapping onsets, detected markers, and mean and SD of asynchrony

This paper continues with an overview of REPP. We then present a series of calibration and behavioral experiments demonstrating key aspects of this technology: temporal accuracy (Experiment 1), test-retest reliability and concurrent validity in the laboratory (Experiment 2), and test-retest reliability in a larger-scale online experiment, as well as methods for ensuring high data quality while minimizing costs (Experiment 3). Finally, we discuss the limitations of REPP and implications for future work on SMS. We release REPP as a free and open-source framework alongside the publication of this paper. Code available in https://gitlab.com/computational.audition/repp

## Overview: REPP

REPP can be organized around five main steps: (i) s*timulus preparation*, (ii) *recording phase*, (iii) *onset extraction*, (iv) *onset alignment*, and (v) *performance analysis* (see Fig. 1b).

REPP takes two inputs: an audio file with the stimulus (e.g., a metronome or a music clip) and a list of the corresponding stimulus onsets. Prior to performing an experiment, the stimulus must be prepared to be used efficiently in the subsequent steps. First, we filter the stimulus to avoid any overlap with the frequency range that will be occupied by participants' tapping responses (e.g., 50–500 Hz). Second, we add custom markers with known temporal locations to the beginning and end of the stimulus, shifting the corresponding stimulus onsets accordingly (see Fig. 1c; yellow boxes). These markers are critical to unambiguously identify the position of the tapping and stimulus onsets in the audio recording, enabling their precise alignment. Thus, we designed the markers to be robustly detected across participants’ hardware and software, including cases of noise-cancellation technologies and noisy recordings. In particular, we generate the markers’ sound by combining a short burst of filtered white noise (50%, filtered to 200–340 Hz) and pure tones (50%, also filtered to 200–340 Hz), and play the resulting sound at nearly maximum volume (see *Custom Markers* in supplementary information for technical details on the markers’ generation procedure).

In the recording phase, REPP uses a free-field recording approach: the prepared stimulus is played through the laptop speakers and the resulting audio signal is simultaneously recorded along with the participant’s tapping response using the built-in microphone (see Fig. 1a). This method returns an audio file where both the audio stimulus and tapping onsets are mixed in the same channel. The next step (i.e., onset extraction) therefore applies signal processing techniques to split the mono recording into separate channels. REPP uses a unique frequency range for each relevant audio element in the recording, including a marker range and a tapping range (see Fig. 1c). The tapping range is determined by the acoustic spectrum of the sound produced by participants’ mode of tapping. In our procedure, participants tap with their index finger on the surface of their laptop, producing a crisp sound with a significant part of its energy between 80 and 500 Hz. Since we have previously filtered the audio stimulus to avoid any overlap with this tapping range, we can efficiently extract the tapping signal from the raw recording by using bandpass filters with cutoff frequencies set to these ranges.

Similarly, we use a specific range to filter and identify the marker locations (i.e., the same range used to generate the marker sound, i.e., 200–340 Hz). In addition, to enhance the markers’ extraction procedure, we compare the amplitude of the filtered markers’ channel against a test channel set to be one octave below the markers’ range, i.e., 100–1070 Hz (Fig. 1c; orange boxes). The test channel is used to compute an estimate of the temporal locations that contain the markers. We then use these estimates to boost the markers’ signal exactly in temporal locations where we found more energy in the markers’ frequency range than in the test frequency range. The rationale here is that tapping sounds will have similar energy within the markers and test channels, whereas marker sounds will have all energy in the markers channel and nearly no energy in the test channel. Thus, by using this principle, we can be sure that the detected signal corresponds to the markers and not to other sources of noise (e.g., tapping signal or background noise). In particular, the resulting signal has enhanced the markers’ amplitude, which helps combat signal attenuation as a result of noise cancelation and noisy backgrounds, effectively increasing the chance of detection only in those areas containing the markers (see *Custom Markers* in supplementary information for technical details on the markers’ extraction procedure, including Figure S7 with examples of successful and failed trials). Next, REPP applies a simple onset extraction algorithm to the filtered markers and tapping channels to detect all samples exceeding a relative threshold (Elliott et al., 2018), returning a vector of extracted tapping onsets and extracted marker onsets.

The last challenge consists of aligning the extracted taps to their position in the audio stimulus. Since the sound card guarantees that all events are recorded with high precision with respect to the beginning of the recording, we can use the first detected marker as a single frame of reference to align the stimulus and tapping response. We use the other markers to assess REPP’s timing performance in each trial and exclude participants with incompatible hardware or software (see *Failing Criteria* in supplementary information). For example, we calculate the markers’ timing error by subtracting the known markers’ locations against the detected markers’ location. This metric is critical to ensure that the timing accuracy of our technology remains high in all trials. Importantly, by relying on the markers’ accurate timing, we do not need to extract the stimulus onsets from the recorded signal, which can be challenging in online studies due to noise-cancellation technologies and interference from other audio elements (e.g., participants' tapping response and background noise). Instead, we use the list of stimulus onsets provided in the stimulus preparation step and, therefore, do not need to extract the onsets from the recorded audio stimulus, minimizing any interference with other elements in the signal processing pipeline. This method allows us to support SMS experiments using “virtual” onsets that are not clearly defined in the audio signal, such as when working with music (Colley et al., 2018; Dannenberg & Wasserman, 2009; Patel et al., 2005; Repp, 2002b). Namely, we can use the list of preregistered onset lists even in the absence of any physical onset in the stimuli. The output of this step is a list of realigned stimulus onsets and realigned tapping onsets. Finally, we calculate several metrics to assess the performance of REPP and measure participants' tapping accuracy, including the mean and standard deviation of the tap-stimulus asynchrony, percentage of detected taps, percentage of detected markers, and markers’ timing error (see Fig. 1d).

## Validation experiments

Three experiments were conducted to validate REPP and show how it can be implemented in online experiments to produce high-quality data. In Experiment 1, we assess the timing accuracy of REPP using an independent calibration system. In Experiment 2, we assess REPP’s test-retest reliability and concurrent validity when measuring individual differences in SMS in the laboratory. Finally, in Experiment 3, we assess the test-retest reliability of REPP with a larger sample of participants recruited online and also provide suggestions to reach high data quality while minimizing recruiting costs. See supplementary information for additional methods and demographic information. All datasets are available as a read-only OSF repository: https://osf.io/r2pxd/

To measure tapping performance, we followed common practices established in previous tapping studies (Repp, 2005). Asynchronies were defined as A_n_ = R_n_ – S_n_, where R_n_ denotes a response onset and S_n_ denotes a stimulus onset in a given tapping trial. We then computed the mean asynchrony and standard deviation (SD) of asynchrony. Throughout the paper, we report the SD of the asynchrony, as it provides a more consistent measurement of tapping accuracy than mean asynchrony. Typically, the mean asynchrony is negative due to a human tendency to anticipate taps by a few tens of milliseconds when synchronizing to an external cue event (Repp, 2005). Mean asynchrony is also more influenced by tapping tasks, production modality, and auditory feedback biases compared to the *SD* of asynchrony. However, we replicated the main results reported in the behavioral experiments (Experiment 2 and 3) when using mean asynchrony (see Figure S8 in supplementary information). Furthermore, we repeated the same analyses using alternative (and more sophisticated) measures of SMS (see *Trialling Alternative Measures of SMS *in supplementary information). These included vector length using circular statistics (Fisher, 1993), a leading model of SMS proposed by Vorberg and Wing (Vorberg & Wing, 1996; Vorberg & Schulze, 2002), including the three hypothesized components (i.e., timekeeper noise, motor noise, and error correction), and lag-1 autocorrelation of the asynchrony and inter-tap interval as alternative measures of error correction.

### Experiment 1: Timing accuracy

This experiment assessed the timing accuracy of REPP by comparing its performance with a ground-truth recording obtained from an independent calibration system. The experiment was divided into three parts. Part 1 and 2 were large-scale validation experiments aimed to extensively test the timing accuracy of the audio stimulus and tapping response, respectively. Part 3 was smaller in terms of the number of data points but aimed to test all components of REPP together (i.e., markers, stimulus, and tapping response).

**Fig. 2:**
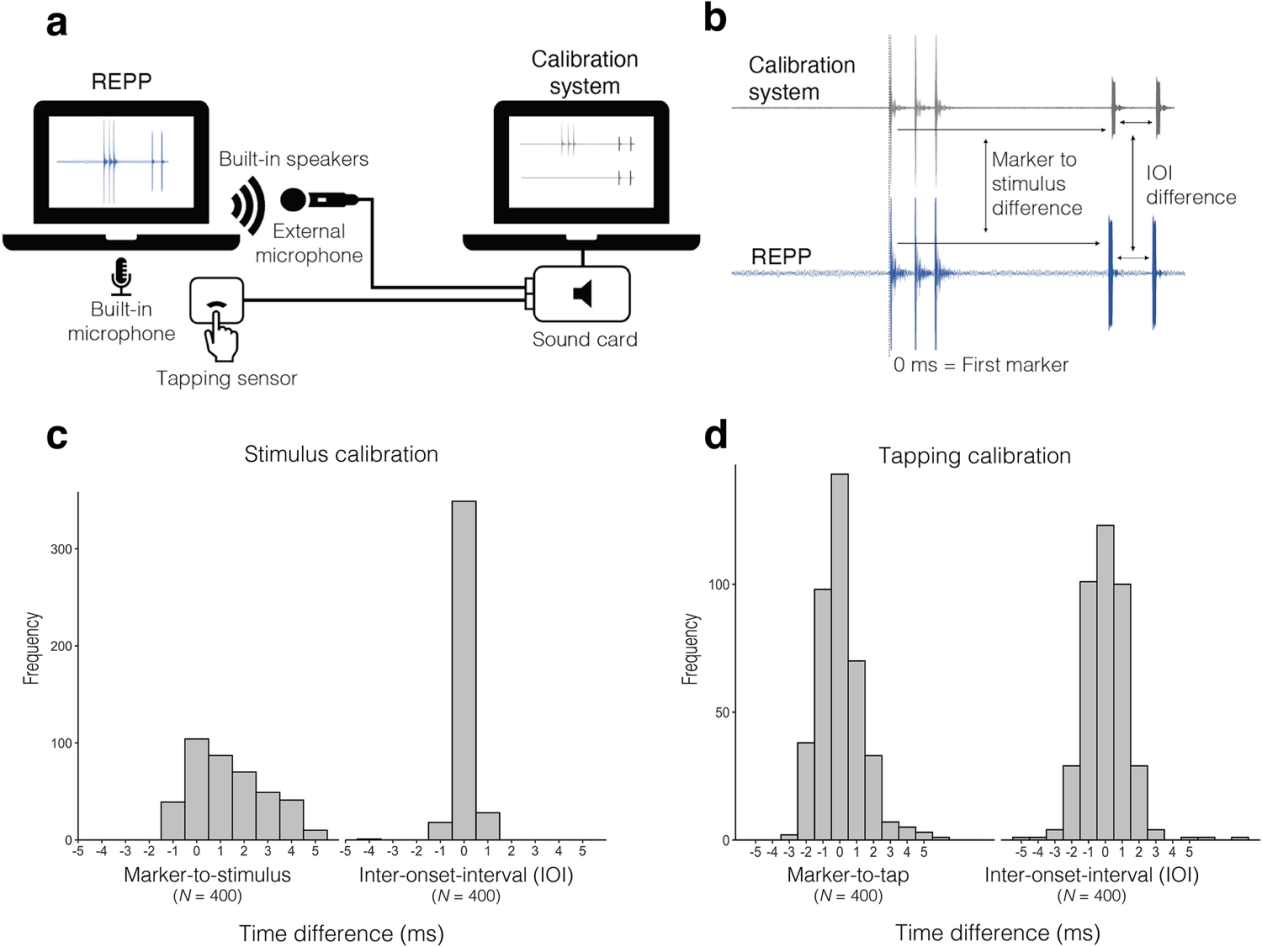
Results of Experiment 1: Timing accuracy. **a** External calibration system used to measure REPP’s timing accuracy. **b** Example of the beginning of a 500 ms IOI trial recorded in the two systems, showing the three marker sounds placed at the beginning of the stimulus and the two first metronome clicks. The figure illustrates the two alternative measures to assess timing accuracy: the difference between the first marker and the stimulus (marker-to-stimulus), and the inter-onset interval. Note that the calibration system has two input channels (external microphone and tapping sensor) but we combine them in the figure for simplicity. **c** Distribution of the difference between the time the stimulus onsets (metronome clicks) were produced and the time they were detected by REPP. *N* refers to the total number of tested onsets. **d** Distribution of the difference between the time the physical taps were produced and the time they were detected by REPP. *N* refers to the total number of tested onsets

REPP’s timing performance was tested in the laboratory against an independent calibration system. This allowed us to measure the cue events (either tapping or stimulus onsets) separately in the two systems, providing an upper bound on inaccuracies of both REPP and the independent calibration system. Based on an established method previously used in our work (Jacoby & McDermott, 2017), we used a calibration system that offers a simple solution for measuring the ground-truth recording of REPP (see Fig. 2a). We tested two variants of this system: one where participants tap on a tapping sensor (part 2) and another where participants tap on the surface of the laptop (part 3). In general, the independent calibration system uses two external synchronized devices to record the stimulus and tapping signals as soon as they are produced by the laptop speakers and finger tap, respectively. Since both the stimulus and tapping signals produce a highly precise sound wave, we can then apply a simple onset extraction algorithm to precisely identify the location of the onsets at the earliest possible moment. To record the audio stimulus (both the markers and stimulus onsets), we directed a Shure SM58 microphone to the laptop speakers being tested. To record the tapping onsets, we used a custom-made tapping sensor device. The sensor consisted of a soft pad with earbuds installed inside (Apple EarPods). The earbuds offer a low-sensitivity microphone that is well-suited to precisely detect touch on the surface of the sensor while being insensitive to external noises and minimizing auditory feedback. The tapping sensor was placed next to the laptops’ built-in microphone to capture the sound of the finger tapping. Both the microphone and tapping sensor were connected to a Focusrite Scarlett 2i2 USB sound card to record the signal on a separate MacBook computer running *Ableton Live 10* Software, saving the resulting recording as a wave file.

To validate the timing accuracy of the audio stimulus (part 1), REPP was programmed to produce 100 isochronous metronome clicks at four different inter-onset intervals (IOIs): 250 ms, 500 ms, 750 ms, and 1000 ms. No finger taps were produced for this part and only the audio stimulus was recorded using the external microphone (Fig. 2a). To validate the timing accuracy of the tapping response (part 2), the same trials were produced and a researcher tapped in time to the clicks, resulting in four trials of 100 taps at four different IOIs: 250 ms, 500 ms, 750 ms, and 1000 ms. The tapping response was recorded using the tapping sensor and the audio stimulus was recorded using the external microphone (Fig. 2a). In the third part, to validate the timing accuracy of all components of the system together (i.e., markers, stimulus, and tapping response), REPP was programmed to produce 20 isochronous metronome clicks at two IOIs: 500 ms and 1000 ms. This time, the researcher tapped on the surface of the laptop in anti-phase, so the stimulus and tapping onsets could be unambiguously distinguished in the recording. Both stimulus and tapping onsets were recorded with the same external microphone. The recording was then separated into three channels (i.e., markers, stimulus, and tapping response) and manually cleaned to only contain the corresponding elements in each channel (e.g., stimulus onsets in the stimulus channel, tapping onsets in the tapping channel).

The results of the timing accuracy analysis in all validation parts are reported in Table 1. The average latency and jitter of REPP was within 2 ms and similarly accurate for all components of the system: markers, audio stimulus, and tapping response. Timing accuracy was computed as the difference between the time the stimulus or tapping onsets were produced (measured using the external calibration system) and the time they were detected by REPP, using the first detected marker as the single frame of reference (Fig. 2b). We used two alternative measurements to calculate timing accuracy: marker-to-stimulus or marker-to-tap (i.e., the interval between the first marker onset and each subsequent onset in the audio or tapping signal), and inter-onset interval (i.e., the interval between onsets). Figure 2c, d shows the distribution of the time difference between the stimulus onsets (part 1) and tapping onsets (part 2) measured in the two systems, confirming that REPP’s timing accuracy is high and consistent.

**Table 1:** Timing accuracy results

Part		*N*	Min-max	Latency (SD)	Min-max	Latency (SD)
			*Relative to first marker*		*Inter-onset interval*	
1 + 2	Markers	40	−.08 to 4.9	1.85 (1.76)	-	-
1	Stimuli	400	−1.3 to 4.9	1.15 (1.61)	−4 to 1	.02 (.39)
2	Tapping	400	−2.8 to 5.9	−.2 (1.37)	−5 to 8	.03 (.13)
3	Markers	10	0 to 1	.58 (.48)	-	-
3	Stimuli	40	−.3 to .9	−.03 (.23)	0 to 1	.04 (.17)
3	Tapping	40	−3.1 to 1	−1.04 (.89)	−4 to 2	−.02 (1.13)

“Part” refers to each validation experiment: part 1 (only stimulus), part 2 (only tapping response), and part 3 (stimulus and tapping response together). *N* refers to the total number of tested onsets.

### Experiment 2: Reliability and concurrent validity

Experiment 1 showed that REPP can measure tapping and stimulus onsets with high temporal accuracy. In Experiment 2, we aimed to examine whether REPP can reliably measure derived psychological quantities such as a particular individual’s tapping accuracy. Specifically, we assessed the test-retest reliability of REPP and compared its performance against a completely independent method: a well-established method previously used in the laboratory to measure SMS with high precision (Elliot et al., 2018).

To assess test-retest reliability, the same group of participants (*N* = 20) performed a short battery of tapping tasks two times in each method, using the following sequence: method 1 (pre), method 2 (pre), method 1 (post), method 2 (post). Half of the participants started the experiment using REPP, whereas the other half started using the independent in-lab method. To assess concurrent validity, we correlated the participants’ overall tapping performance in the two methods. Participants completed the experiment in a quiet testing room with two tables, one for each method.

**Fig. 3:**
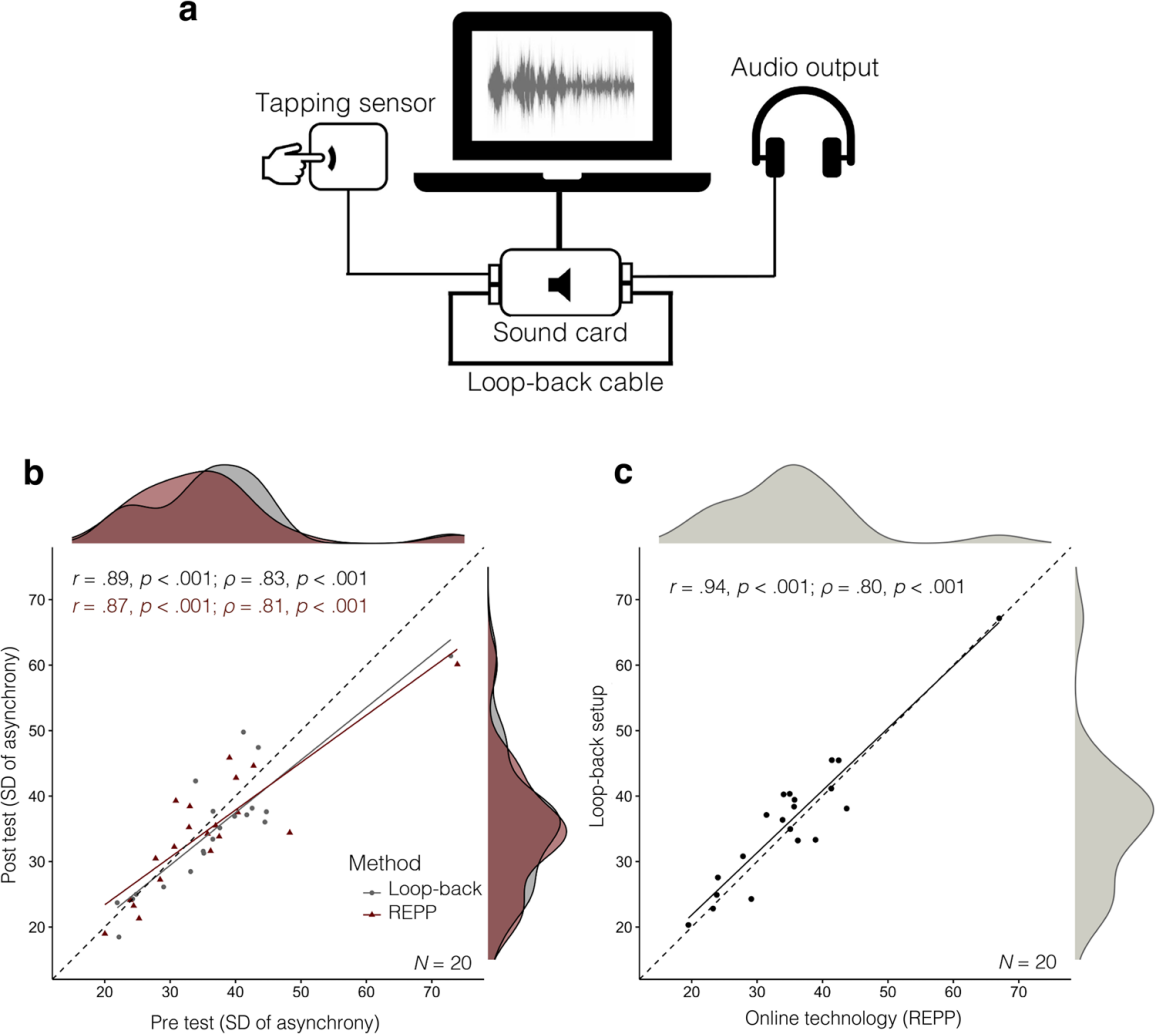
Results of Experiment 2: reliability and concurrent validity. **a** Loop-back setup: independent in-lab method using a loop-back cable to measure participants’ tapping asynchronies with high temporal fidelity (Elliot et al., 2018). **b** Test-retest reliability in the two methods. **c** Concurrent validity: correlation between the overall tapping performance measured in the two methods

The independent in-lab method consisted of a loop-back setup to measure participants’ asynchronies with high temporal fidelity (see Fig. 3a). This method has been extensively used in the laboratory and field research on SMS (Elliot et al., 2018; Jacoby & McDermott, 2017; Jacoby et al., 2021). The loop-back setup consists of a cable connected to a sound card to simultaneously record the input signal from the headphones and the output signal from a tapping sensor with nearly zero latency. We used professional headphones (i.e., Sennheiser HD 280 Pro headphones) to deliver the stimulus, and the custom-made tapping sensor described in Experiment 1 to record participants’ tapping response with high precision. Both the headphones and tapping sensor were connected to an external sound card (Focusrite Scarlett 2i2 USB) using the loop-back setup described above (Fig. 3a). The sound card was connected to a MacBook via USB. The tapping tasks were implemented using MATLAB, mirroring the experimental procedure used in REPP. To detect the stimulus and tapping onsets at the earliest possible moment, we used the same onset extraction algorithm described in Experiment 1 (Jacoby & McDermott, 2017).

The materials and experimental procedure were identical in the two methods (see *Instructions* in supplementary information). Before starting the main tapping task, participants performed a practice phase to get familiar with each method (see *Practice Phase* in supplementary information). The main tapping tasks consisted of a short battery of tapping trials (approximately 8–10 minutes long) using two common paradigms in the tapping literature (Repp, 2005; Repp & Su, 2013): isochronous tapping and beat synchronization to music. The isochronous tapping consisted of four 30-second-long trials of isochronous tapping to a metronome sound (two with IOIs of 800 ms and two with the IOIs of 600 ms). The presentation order was fixed, using the following sequence: 800 ms, 600 ms, 800 ms, and 600 ms. The beat synchronization task consisted of four 30-second-long excerpts of music from two distinct music genres with different style, tempo, and tapping difficulty, also with fixed order of presentation (see *Beat Synchronization Task *in supplementary information).

The results of Experiment 2 are plotted in Fig. 3. Tapping performance was measured using the SD of the tap-stimulus asynchrony (see supplementary information for the results of the same analysis using alternative measures of SMS). To examine test-retest reliability, an aggregated performance score was calculated for each participant in each test (pre and post) and method by averaging their tapping performance in the two tapping tasks (i.e., isochronous tapping and beat synchronization to music). The test-retest correlation in REPP was high (*r* = .87; *ρ =* .81) and similar to the one achieved by the independent loop-back setup (*r* = .89; ρ *=* .83; Fig. 3a). We further examined test-retest reliability by calculating the intraclass correlation coefficient (ICC; Shrout & Fleiss, 1979). Following the recommendations of Koo and Li (2016), ICC estimates and their 95% confidence intervals were calculated based on single-rating, absolute-agreement, 2-way mixed-effects models (ICC3). We found a good ICC in both REPP (ICC = .86, 95% [.72, .93]) and the loop-back setup (ICC = .89, 95% [.77, .95]). This ICC is comparable to the values reported in previous work assessing the test-retest reliability of similar rhythmic production tasks (Bégel et al., 2018). Moreover, an ANOVA confirmed that participants’ mean tapping performances were similar across test-retest conditions and tapping tasks (all *p*-values > .05). Finally, we found that REPP has a high concurrent validity (*r* = .94 and *ρ =* .89), as indicated by the correlation between the overall tapping performances (averaging over both test and retest) measured by the two methods (Fig. 3b). In conclusion, the converging evidence of these analyses is that REPP produces reliable estimates to measure individual differences on SMS in a way that is consistent with the results produced by a completely independent method.

### Experiment 3: Online demonstration

Having demonstrated that REPP achieves high temporal accuracy (Experiment 1) and test-retest reliability in the laboratory (Experiment 2), this experiment aimed to show that the technology can work in practice in an online setup that is similar to a large-scale data collection process. We also provide suggestions to ensure high data quality while enabling realistic data collection, in particular concerning pre-screening tasks and feedback based on recording quality and tapping performance. Participants were recruited from Amazon Mechanical Turk (see *Participants* in supplementary information) and performed the same battery of tapping tasks used in Experiment 2. In a total of six experimental batches (8 to 10 hours each), we collected valid tapping data for 226 participants.

We used two pre-screening tasks to ensure high data quality while minimizing recruiting costs (see *Pre-screening Tests *in supplementary information). First, we used an attention test to determine whether participants were paying attention to the instructions. Participants who failed the attention test were excluded from the experiment. Second, we used a recording test to determine whether participants were using hardware and software that were not compatible with REPP, such as malfunctioning speakers or microphones, or the use of strong noise-cancellation technologies. Participants who did not pass the recording test were also excluded from the experiment. To assess the efficacy of the recording test in comparison to the attention test on its own, we only used the recording test in half of the participants.

Before the main tapping tasks, participants were instructed on several key aspects concerning the proper functioning of REPP, including instructions about the technical requirements and tapping procedure for the experiment, a volume calibration test, and a tapping calibration test (see *Instructions* in supplementary information). Next, participants undertook a practice phase consisting of four trials of isochronous tapping to a metronome sound (see *Practice Phase* in supplementary information). After completing the practice phase, the four audio recordings were analyzed in real time using a failing criteria designed to identify and fail trials where participants used incompatible hardware and software, or where participants did not tap as indicated in the instructions (see *Failing Criteria* in supplementary information). Those participants who failed two or more trials were excluded from the experiment. After the practice phase, participants started with the main experimental task, which consisted of the same battery of tapping tasks employed in Experiment 2 (i.e., four trials of isochronous tapping and four trials of beat synchronization to music, 30 seconds long each). Participants repeated the same battery of tapping tasks a second time in order to measure test-retest reliability.

**Fig. 4:**
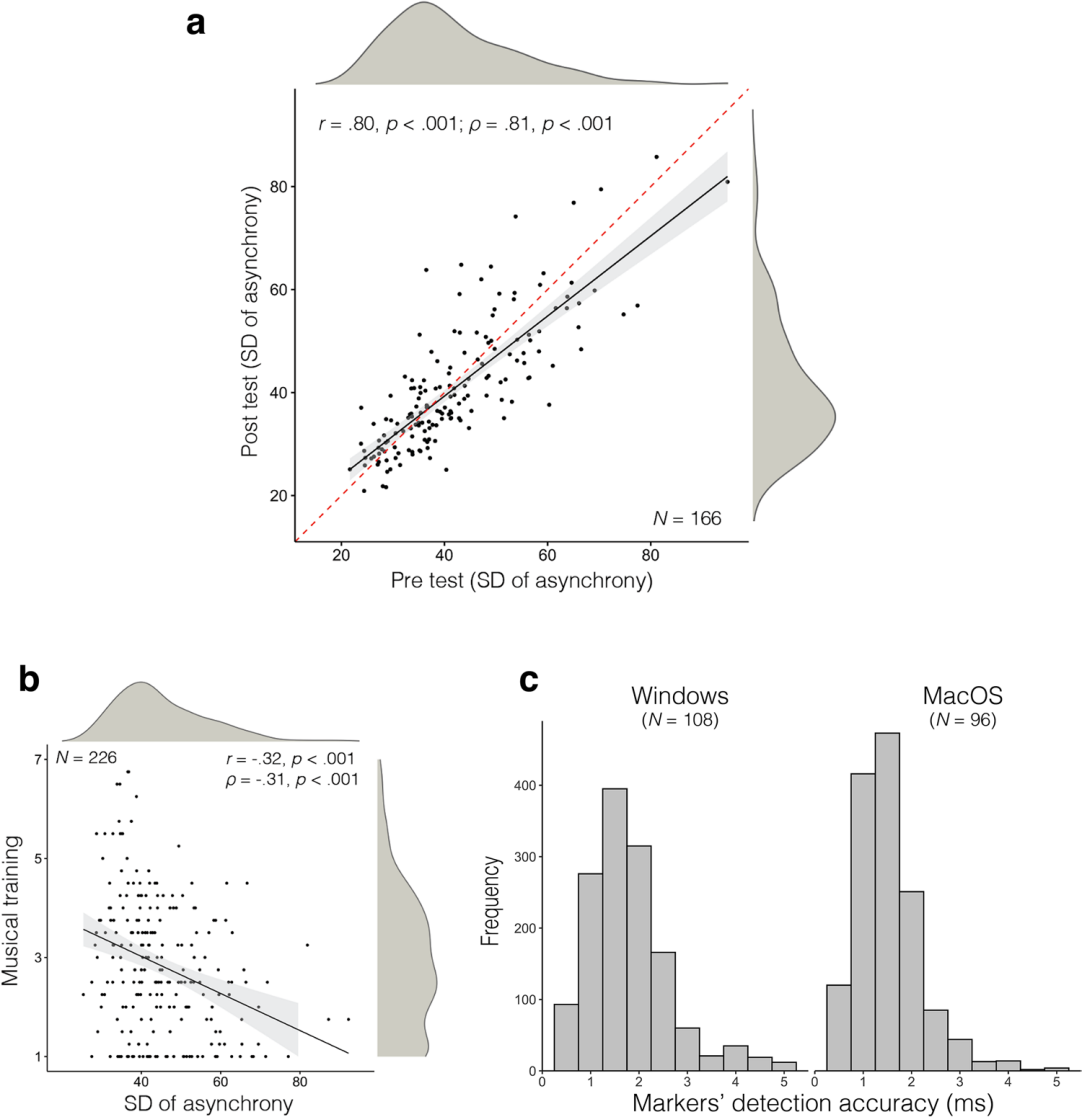
Results of Experiment 3: Online demonstration. **a** Test-retest reliability of REPP when measuring participants’ tapping performance online. **b** Convergent validity: correlation between overall tapping performance and participants’ musical training. **c** Estimated markers’ error in Windows and macOS computers. *N* indicates the number of participants using each operating system, but we plot the data in all tapping trials (2814 in total)

The results of Experiment 3 are visible in Fig. 4 (see supplementary information for the same analysis using alternative measures of SMS). For measuring test-retest reliability, we only consider participants who provided at least one valid tapping trial for each stimulus in each tapping task and test-retest condition (*N* = 166). Test-retest analyses were performed using the same procedure described in Experiment 2. Results indicated a high test-retest correlation when using REPP to measure participants’ tapping performance online (Fig. 4a; *r* = .80 and *ρ* = .81), also confirmed by an intraclass correlation analysis (ICC = .82, 95% [.77, .86]). Moreover, participants’ tapping performance was similar across test-retest conditions in the two tapping tasks, as indicated by two paired-samples *t*-tests with test condition as the independent variable and tapping performance in each tapping task as dependent variables (all *p*-values > .05). As a measure of convergent validity, we further examined whether participants’ tapping performance was related with their self-reported levels of musical training. We calculated an aggregated tapping performance score (averaging over test and retest in the two tapping tasks) for all participants who provided good tapping (*N* = 226). Musical training was measured using a reduced version of the Gold-MSI musical training factor (Müllensiefen et al., 2014). Replicating a recurring finding in the literature (e.g., Niarchou et al., 2021; Repp, 2010; Thompson et al., 2015), we found a significant negative correlation (*r* = -.32, *p* < .001) between tapping variability and self-reported musical training (Fig. 4b), indicating that participants with more musical training were better at synchronizing to an external beat. To explore the robustness of our technology across operating systems and laptop models, we compared the markers’ detection accuracy (i.e., the delay between the known marker locations and the detected marker onsets) across the two most common operating systems, Windows and macOS (Fig. 4c). Overall, trials recorded in macOS computers achieved slightly better temporal accuracy (*M* = 1.48, *SD* = .68) than trials recorded in Windows (*M* = 1.74, *SD* = .85), *t*(2812) = 9.36, *p* < .001. A small difference in this direction is not surprising: macOS computers are typically better equipped for delivering sound and recording audio than Windows computers, which also tend to exhibit greater variability in hardware.

## Discussion

SMS is an active area of research with a long history of tapping experiments performed in the laboratory (Repp, 2005; Repp & Su, 2013). However, it currently lacks a robust method to precisely measure participants’ asynchronies in online experiments. In this paper, we presented REPP, a cross-platform solution for online SMS experiments that achieves high temporal accuracy and reliability while also being practical in terms of large-scale data collection. We release this technology as a free and open-source framework alongside this paper.

We validated REPP in three experiments. We first demonstrated that it achieves high temporal accuracy using an independent calibration system (Experiment 1). Based on the ground-truth recording, we estimated the average latency and jitter of REPP to be within 2 ms and similarly accurate for all elements in the system (i.e., markers, audio stimulus, and tapping response). In a laboratory experiment (Experiment 2), we then compared the test-retest reliability of REPP with a completely independent method that uses specialized equipment (i.e., a loop-back setup) to measure SMS with optimal temporal fidelity. We found that our technology achieves a high test-retest reliability (*r* = .87, *ρ* = .81, ICC = .86) that is equivalent to the reliability obtained by the independent in-lab method (*r* = .89, *ρ* = .83, ICC = .89). By correlating the overall tapping performance measured in the two methods, we also found that REPP has a high concurrent validity (*r* = .94 and *ρ* = .80). Finally, we showed that REPP can work in practice in an online setup that is similar to a large-scale data collection process (Experiment 3). In particular, we confirmed that REPP has a high test-retest reliability using a larger sample of participants recruited online (*N* = 166; *r* = .80, *ρ* = .81, ICC = .82). We also provided suggestions of tapping instructions and pre-screening tests to ensure high data quality in online experiments while minimizing recruitment costs (see supplementary information). Together, these experiments demonstrate that our technology is well-equipped to support a wide variety of SMS experiments using standard hardware and software available to most online participants.

A core aspect of REPP is the ability to analyze participants’ recordings in real time, making it possible to monitor experiments online and provide feedback almost instantly. We used a software testing tool^1^ to reliably estimate the speed of processing of this technology under different analysis conditions (for each condition, we repeated the same analysis a thousand times)^2^. We found that running the analysis when plotting is disabled leads to the fastest performance. For example, the average running time (Q1–Q3) to analyze a 15-second-long recording is 0.66 seconds (0.64–0.67), whereas the average running time (Q1–Q3) for a 30-second-long recording is 1.46 seconds (1.42–1.49). When plotting is enabled (generating and saving plots for each analysis), the average running time (Q1–Q3) is 3.84 seconds (3.75–3.89) for a 15-second-long recording and 5.55 seconds (5.29–5.81) for a 30-second-long recording. This is mainly due to the slow implementation of the python package *Matplotlib*^3^ that we used for plotting. In case this is important, experimenters can avoid plotting (as it is only used for debugging) or consider implementing a more efficient plotting library (such as *VisPy*^4^ or *PyQtGraph*^5^). Overall, these results show that REPP is suitable to process participants’ recordings in real time, enabling researchers to filter participants based on performance or provide trial-to-trial feedback. The ability to rapidly process participants’ performance is also crucial to enable more complex SMS paradigms in online settings, such as iterated tapping experiments where new stimuli are generated on the fly based on previous tapping responses. In Jacoby et al. (2021), for example, we successfully adapted an iterated tapping paradigm using REPP in a large-scale online tapping experiment conducted in the US, India, and Brazil.

REPP currently has some limitations. First, it does not support all functionalities that are offered in many in-lab methods (e.g., *FTAP*, *Max-MSP*, or *MatTAP).* For example, REPP currently does not support real-time response feedback (Mates & Aschersleben, 2000; Finney & Warren, 2002). A possible solution would be to play the real time feedback with a JavaScript audio process; this feedback may have compromised accuracy, but at least the feedback signal could be recorded and monitored with a variant of our technology. Developing this approach would however require significant additional work. Second, REPP currently only works with laptop speakers. We see great potential in extending our technology to mobile devices and tablets as well. This would not only make REPP more accessible, but it would also open the possibility to run SMS experiments while in motion, such as walking or dancing. Combining REPP with features available in most mobile devices, such as multi-touch screens or accelerometers, can also enable new ways to collect data and modes of interaction. Third, REPP relies on a stimulus preparation step that filters the audio stimulus to remove lower frequencies that would otherwise interfere with other aspects of the signal processing pipeline, such as the analysis of participants’ tapping response. This procedure decreases the perceived quality of complex auditory stimuli, such as music, and it could therefore compromise its ecological validity. We encourage future research to test this in the lab, for example, by comparing participants’ tapping accuracy in a beat synchronization task using music stimuli filtered at different frequency ranges. Nevertheless, it is worth noting that in the two behavioral experiments conducted here we did not receive any complaints from participants regarding the quality of the music stimuli. More importantly, participants’ tapping performance in the online beat synchronization task (using filtered music) was within an expected range (Experiment 2, *N* = 166, SD of asynchrony = 42.68; SD = 15.82) and similar to the their performance in the online isochronous task tapping using an unfiltered metronome sound (Experiment 2, *N* = 166, SD of asynchrony = 40.68; SD = 11.98), also supported by a large correlation between participants’ tapping performance in the two tasks (*r* = .58, *ρ =* .59, all *p*-values < .001). This suggests that the filtering step does not drastically impair participants ability to synchronize to a musical beat when using REPP in online SMS experiments.

Moreover, we learned that collecting good tapping data over the internet can necessitate a high exclusion rate, at least when using a large-scale recruiting strategy via Amazon Mechanical Turk. In Experiment 3, for example, a total of 727 participants began the online task. This includes anyone who accepted the experiment regardless of their intentions to take the task seriously or whether they met the technical requirements to provide good tapping data. Thus, we used a practice phase to familiarize participants with the task and exclude cases who could not provide good tapping data in the majority of trials. Note that we used relatively strict failing criteria to exclude trials based on whether the signal could be correctly recorded and whether participants produced a minimally acceptable number of tapping responses (see *Failing Criteria* in supplementary information). A total of 226 participants (31%) passed the practice phase and were able to provide good tapping data. The remaining 483 participants (69%) were excluded from the experiment and comprised a mix of fraudulent participants (e.g., computer bots or non-serious responders) and participants that did not meet the technical requirements of REPP, such as poor internet connection or incompatible hardware and software. Common sources of failure included participants’ behavior, such as not tapping at all, using desktop computers without built-in microphones, or performing the experiment with headphones instead of the laptop speakers. We also noticed that many participants did not follow the instructions to eliminate background noise, such as music or speaking, resulting in noisy recordings. An additional problem was the usage of remote desktops, which may be employed by some participants to alter their geographical reported location. Since the remote desktop will open a microphone that is not physically connected to the computer of the participant, the technology is not able to record any signal. Furthermore, there were several cases where the technology failed due to laptops with low quality or malfunctioning speakers. The same occurred in laptops with strong noise-cancelling technologies, where the marker sounds are suppressed and cannot be detected in the signal. This last issue requires further investigation, as in theory it is possible to turn off noise cancellation manually in most devices, but the way to do so changes in different computer models and brands.

Naturally, the more demanding the online tasks, the higher the exclusion rate. In previous work, we found exclusion rates of only about 10% in Amazon Mechanical Turk experiments with minimal technical requirements, such as when using visual rating scales in the browser (Harrison et al. 2020). However, the exclusion rate increases when the experiment becomes technically more demanding. For example, a pre-screening test that requires participants to wear headphones to perform an auditory perception task produces an estimated exclusion rate of 36% (Wood et al., 2017), whereas performing online research with computer webcams can necessitate an exclusion rate of about 40% (Tran et al., 2017). In language production experiments that require participants to record themselves using a microphone to extract voice onset latencies, the exclusion rate can be around 60% (Vogt et al., 2021). Thus, the exclusion rate of REPP (~60–70%) is not unexpected when using a large-scale recruiting strategy via Amazon Mechanical Turk, as it is technically more demanding than previous paradigms. In particular, REPP can only work in SMS experiments when the marker sounds can be detected with high millisecond-level precision and participants take the task seriously (i.e., tapping with their index finger on the surface of their laptop in time to an auditory stimulus). A high exclusion rate is not particularly problematic when using online recruitment systems with large pools of active participants, but other modes of recruiting may require different strategies, such as when recruiting participants from special populations or using internal university systems. In these cases researchers can significantly reduce exclusion rates by using more relaxed failing criteria and taking more time to support participants and ensure they follow the instructions and meet the technical requirements. REPP can also be used in laboratory studies and field research with an exclusion rate of effectively 0%, as shown in Experiment 2.

Since exclusion rates may be high when using technical demanding tasks in online recruiting systems, such as Amazon Mechanical Turk or Prolific, we strongly recommend the use of pre-screening tests to determine whether participants will take the experiment seriously and meet the technical requirements to provide good tapping data. In Experiment 3, we analyzed the efficacy of two pre-screening tests, an attention test and a recording test (see supplementary information for a full description). We defined the exclusion rate of the pre-screening tests in terms of the proportion of participants who successfully passed the practice phase. Accordingly, we found that when using both pre-screening tests, 68% of the participants were able to pass the practice phase and deliver good tapping data in the experiment. In contrast, when only using an attention test without the recording test, the percentage was 31%. Thus, adding a recording test at the beginning of the experiment reduces the costs of recruiting participants online by nearly half. Such practices can also help maintain a good reputation in the online community; for example, both the attention test and recording test help eliminate a large proportion of the failure rate that comes from fraudulent participants, including computer bots and non-serious respondents (Crump et al., 2013). In addition to pre-screening tests, we encourage the use of data quality checks to monitor participants’ performance throughout the experiment. REPP computes several metrics to check the quality of a given recording, such as the number of detected markers or the time error between the known locations of the markers and the detected onsets, which provides a reliable measure of timing accuracy at the trial level. We can also know how well participants are tapping by computing the ratio between the number of detected onsets and the number of stimulus onsets. Using these metrics, we can provide feedback each time a participant completes a tapping trial, indicating whether their recording quality is sufficiently good and if it is not, suggesting ways to improve it for the subsequent trials (we apply this strategy in the practice phase of Experiment 3). We encourage future research to explore these options further in order to increase recruitment efficiency, such as providing more detailed feedback after tapping trials or nudging participants online to meet the technical requirements.

It is worth noting that REPP can be easily extended to support online experiments requiring precise timing of tapping response without any synchronization to an external stimulus. For example, to perform unconstrained finger tapping paradigms where participants are not given an external stimulus but instead are asked to tap at their preferred rate (e.g., Collyer et al., 1994), or imitation experiments in which participants replicate a rhythm from memory (e.g., Ravignani et al., 2016). In these cases, researchers can skip the stimulus preparation and onset alignment steps, and simply use the parts of the pipeline that are directly related to the onset extraction procedure. We have successfully explored these options using a simplified method for several experiments that do not require stimulus-response synchronization, and support this variant in the code package published with this paper. Another simple extension of REPP is from finger tapping to other modes of production, including clapping, tapping on a table, or speech. We noticed that our technology works well for clapping or tapping on a table, but adapting it to spoken utterances may require a modification to the parameters of the signal processing pipeline (see Experiment 6 in Jacoby & McDermott, 2017). Potentially, our technology could also be used to support online experiments requiring precise timing in domains other than rhythm perception and production. This includes any experiment measuring reaction times to auditory stimuli, such as auditory lexical decision tasks (e.g., Blumstein et al., 1982; Goldinger, 1996), priming paradigms using spoken words (e.g., Radeau et al., 1998), sounds (e.g., Schön et al., 2010), or music (e.g., Bharucha & Stoeckig, 1986, 1987), and experiments on temporal processing using time interval production tasks (e.g., Jazayeri & Shadlen, 2010).

We hope REPP plays a major role in improving the efficiency, scalability, and reach of SMS research. Finding new ways to allow online data collection has become particularly important during the COVID-19 pandemic, with many researchers unable to run experiments in the laboratory. Supporting online experiments on SMS will also significantly reduce the time and resources that researchers usually spend to recruit and test participants in the laboratory. For instance, recruiting 20 participants in the laboratory (Experiment 2) took us approximately one week of work, whereas in the online version of the same experiment (Experiment 3) we recruited 20 valid participants in about 2 hours. Importantly, online experiments allow for the collection of significantly larger and more diverse samples of participants, both demographically and culturally. This is crucial for moving away from the relatively restricted and small samples of university students that laboratory studies tend to rely on (Henrich et al., 2010). Moreover, since online SMS experiments are more accessible and easier to share, they can improve research diversity and collaboration worldwide, an important challenge in today’s cognitive science (Barret, 2020; Savage et al., 2021). Finally, by increasing the reach, scalability, and speed of data collection, online experiments open new avenues for research on SMS that would be nearly impossible in the laboratory. For example, very large-scale studies with hundreds of participants to study individual differences on SMS in the general population (Niarchou et al., 2021), or cross-cultural experiments that include a diverse sample of participants from around the world (Jacoby et al., 2021). Online tapping experiments can also allow for large scale simulated experiments on cultural transmission that were previously conducted only with small cohorts of lab participants (Raviginani et al. 2016). Similarly, the ability to collect large tapping datasets online can also help increase our understanding of the role of SMS in the context of various neurodevelopmental disorders, including attention deficit hyperactivity disorder (Noreika et al., 2013), dyslexia (Colling et al., 2017; Thomson & Goswami, 2008), and Parkinson’s disease (Bieńkiewicz & Craig, 2015).

## Supplementary Information

ESM 1 (PDF 2.02 mb)

## Footnotes

1 https://pypi.org/project/pytest-benchmark/

2 We used a MacBook Pro (macOS Catalina version 10.15.7), with an Intel processor 2.3 GHz 8-Core Intel Core i9, and Python 3.9.4.

3 https://matplotlib.org/

4 https://vispy.org/

5 https://www.pyqtgraph.org/

## Declarations

### Code availability

We release REPP as a free and open-source framework alongside the publication of this paper. Code available in https://gitlab.com/computational.audition/repp
