# Supplementary material for: REPP: A robust cross-platform solution for online sensorimotor synchronization experiments
Source: Behav Res Methods. 2022 Feb 11;54(5):2271–85. doi: 10.3758/s13428-021-01722-2 (PMC8853279; doi:10.3758/s13428-021-01722-2)
Supplement: Supplementary file 1 — (PDF 2.02 mb) [file 13428_2021_1722_MOESM1_ESM.pdf]

Supplementary Information

REPP: A Robust Cross-Platform Solution for Online Sensorimotor Synchronization Experiments

Manuel Anglada-Tort, Peter M. C. Harrison, and Nori Jacoby

Participants

Experiment 2

Participants were recruited using the internal database of the Max Planck Institute for Empirical Aesthetics (Frankfurt, Germany), with the requirement that they were at least 18 years old and had a basic understanding of English. All participants provided consent in accordance with the "Approval of Research Projects Using Standard Procedures" protocol of the Max Planck Society Ethics Council (revised version 2017). The experiment took about 1 hour and the reimbursement was 14 €. A total of 20 participants (10 female, 10 male), aged 20-59 (M = 30.05, SD = 11.88) took part in the experiment

Experiment 3

All participants were recruited online using Amazon Mechanical Turk. All online participants provided consent in accordance with the Max Planck Society Ethics Council approved protocol (application 2018-38). We asked for five requirements in order to take part in the experiment: (i) participants must be at least 18 years old, (ii) participants must be fluent English speakers, (iii) participants must use a laptop to complete the experiment (no desktop computers allowed), (iv) participants must use an up-to-date Google Chrome browser (due to compatibility with *PsyNet*), and (iv) participants must be sitting in a quiet environment (to ensure that their tapping could be recorded precisely). In addition, to help recruit reliable participants, we only recruited participants with a 95% or higher approval rate on previous tasks on Amazon Mechanical Turk. Participants were paid at a US $9/hour rate according to how much of the experiment they completed (e.g., if participants failed a pre-screening task and left the experiment early, they were still paid proportionally for their time). The complete experiment took approximately 20-25 minutes.

A total of 226 participants provided valid tapping data in at least one trial, having already excluded all those who failed the pre-screening tests or the practice phase. For those participants who reported demographic information, ages ranged from 19 to 77 (M = 35.9, SD = 11.9), and 46% identified as female (54% as male).

Implementation

We implemented REPP as a *Python* package. In all experiments, REPP was integrated into our in-house system to perform behavioral experiments - *PsyNet* (Harrison et al. 2020). This system is based on the Dallinger framework[1] for hosting and deploying experiments. Participants interact with the experiment via a web browser, which communicates with a back-end Python server cluster responsible for organizing the experiment and communicating with REPP. This cluster can run using a local webserver (for in-lab experiments) or by a cloud Platform as a Service such as Heroku (for online experiments). Currently, *PsyNet* is only supported by Google Chrome.

[1] https://github.com/Dallinger/Dallinger

Procedure

Participants were informed that the experiment can only be performed using laptop speakers. We then used a volume calibration test so participants can adjust the volume of the speakers to a level that is sufficiently good to be detected by the microphone. In the volume calibration page, we play an audio stimulus through the speakers and record the signal with the built-in microphone, using a sound meter to visually indicate whether the level was appropriate or not (see Figure S1 for a screenshot of the volume test). Participants were then instructed about how to tap on their laptop in a way that is compatible with REPP and also feels natural to them: "Tap on the surface of your laptop with your index finger (e.g., do not tap on the keyboard or tracking pad, and do not tap using your nails or any object)". Here we used a tapping calibration test to ask participants to practice tapping in the required way and test if the microphone could detect their signal, also using a sound meter to give feedback visually (see Figure S1 for a screenshot of the tapping test). In cases where the signal was too low, participants were indicated to tap in different locations of the laptop or try to tap louder.

Pre-screening Tests

When running experiments online, it is important to ensure participants follow the instructions and perform the task as required (e.g., Clifford & Jerit, 2014; Crump et al., 2013). In addition, REPP has several technical requirements that participants must meet in order to provide valid tapping data. To address this, we used two pre-screening tests in the online experiments reported in this paper (Experiment 3): an attention test and a recording test.

Attention Test. The attention test was used to determine whether participants were paying attention to the instructions or not (see Figure S2 for a screenshot of the attention test). The test consisted of two pages that could only be passed if a participant carefully read the instructions. The attention test was presented at the beginning of the experiment after asking for general demographic information. In our implementation, participants who failed the first page in the attention test were excluded from the experiment, whereas the second page was used for post-hoc quality assessment (we did not exclude participants based on failure to answer correctly in the second page).

Recording Test. The recording test was used to determine whether participants were using hardware and software that did not meet the technical requirements of REPP, such as malfunctioning speakers or microphones, or the use of strong noise-cancellation technologies (see Figure S3 for a screenshot of the recording test). The recording test was used at the beginning of the experiment, after providing general instructions with the technical requirements of the experiment. Thus, this test can also be used as an attention test, as participants must follow the given instructions (e.g., accept the enabling of the microphone in the browser, unplug any headphones or wireless devices, turn up the volume of the computer) in order to successfully pass the test. For example, bots that click randomly on the screen would

naturally not be able to complete these steps. The recording test consisted of a recording page that played a test stimulus with six marker sounds. The markers were recorded with the laptop's microphone and analyzed using the signal processing pipeline. During the marker playback time, participants were supposed to remain silent (not respond). In our implementation, we used two recording trials. Those cases in which all marker sounds could not be detected in one of the two recording trials were excluded from the experiment.

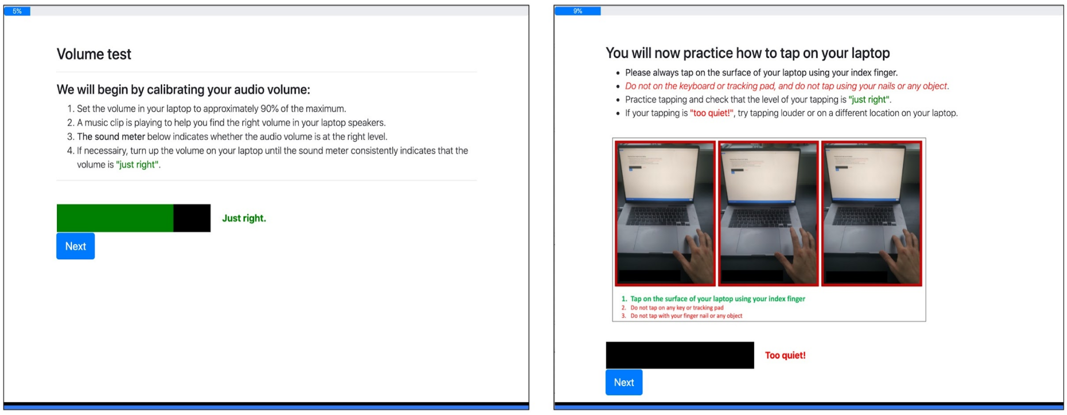

Figure S1. Volume and tapping calibration tests using sound meters to provide visual feedback

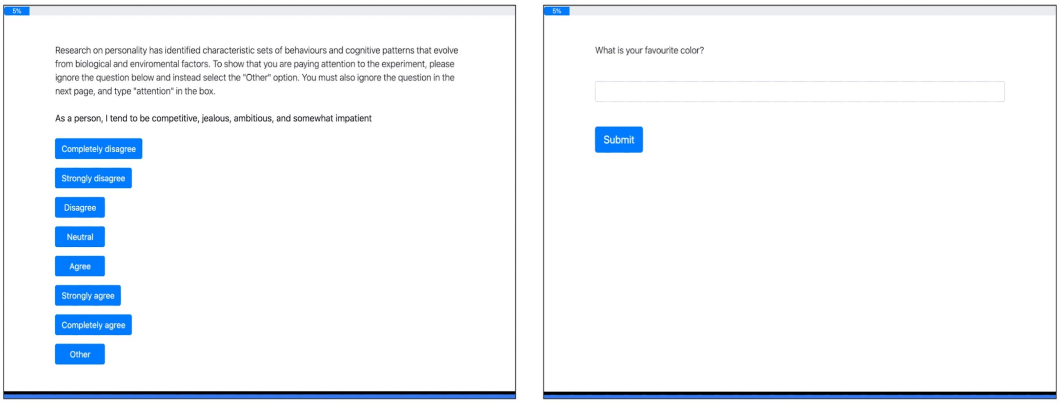

Figure S2. Attention test to determine whether participants follow the instruction in online experiments

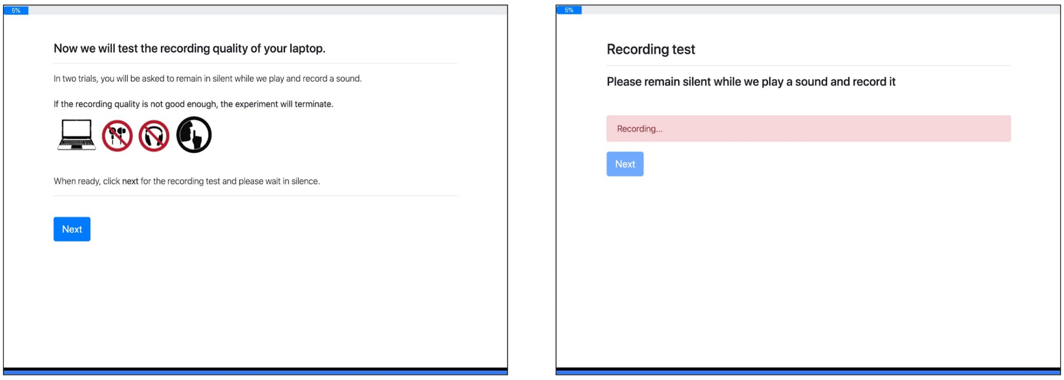

Figure S3. Recording test to determine the performance of REPP in online experiments

Practice Phase

In Experiment 2 (laboratory), the practice phase consisted of two trials of isochronous tapping to a metronome sound (each trial was 20 seconds long, one with IOIs of 800 ms and the other with IOIs of 600 ms). Participants performed a practice phase the first time they used each method, one for REPP and one for the independent in-lab method. A researcher was present during the practice phase to provide feedback on participants' practice trials.

In Experiment 3 (online), the practice phase consisted of four trials of isochronous tapping to a metronome sound (two with IOIs of 800 ms and two with IOIs of 600 ms, 20 seconds long each). Moreover, the recording of the first practice trial was analyzed in real time to provide feedback based on the quality of the audio and tapping signal (using the *Failing Criteria* described below). If the signal of the recording did not pass the failing criteria, participants were reminded of the instructions and were able to continue with the other practice trials. At the end of the practice phase, all trials were analyzed online using the same procedure and participants who failed two or more trials were excluded from the experiment. All participants were compensated proportionally to the time spent in the experiment, even if they failed the screening tests or practice phase. Figure S4 shows an example of the instructions and tapping trial given in the practice phase of Experiment 3. In each trial, to help participants only tap when the stimulus was played (and remain silent when the marker sounds were presented), we visually indicated on the screen when to start and when to stop tapping.

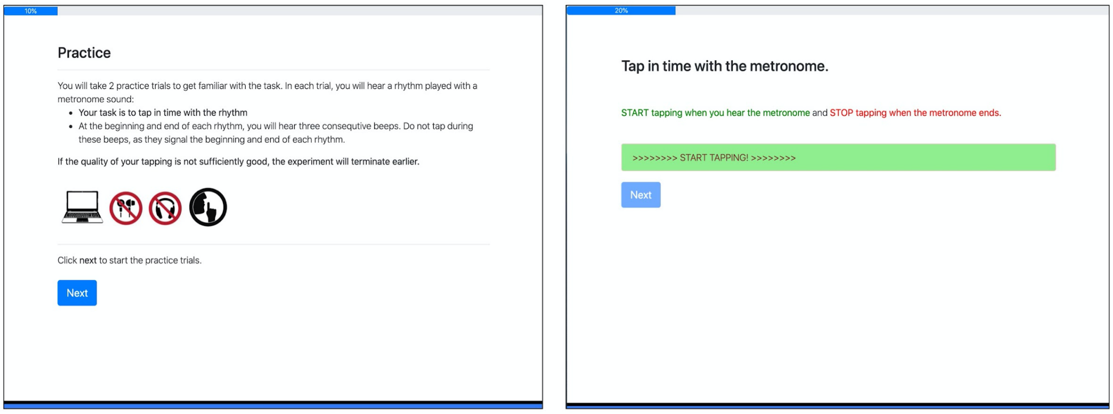

Figure S4. Instructions and tapping trial in the practice phase

Beat Synchronization Task

In the beat synchronization task (Experiment 2 and 3), participants were instructed to tap in time to with the beat until the music ends (Figure S5 shows a screenshot of the instructions for the beat synchronization task). As commonly used in this type of paradigm, to help participants find the beat, a metronome marking the beats in the first 11 seconds of the clip was added to the stimulus. To motivate participants to continue tapping accurately until the end of the clip we also added three more metronome beats to the end of the recording. Thus, to calculate participants' tapping performance in this task, we only analyzed the stimulus onsets when the metronome was not played. The materials of the beat synchronization task consisted of four 30-second-long excerpts of music from two distinct music genres with different style, tempo, and tapping difficulty: track 1 (“You're the First, the Last, My Everything” by Barry White) and track 2 (“Le Sacre du Printemps” by Stravinsky). The presentation order was fixed, namely: track 1, track 2, track 1, and track 2. The musical excerpts were taken from the MIREX 2006 Audio Beat Tracking database, which also provides annotations for beat locations given by listeners who tapped along to the music (McKinney et al., 2017). Based on these annotations, we identified the target beat locations from those consistently produced by the annotators using the following procedure: First, we performed kernel density estimation with a kernel width of 20 ms to find the mode of participants' responses in any given time. Second, we locate the peaks of the probability density to find all onset locations in the music by identifying local maxima in the kernel density function.

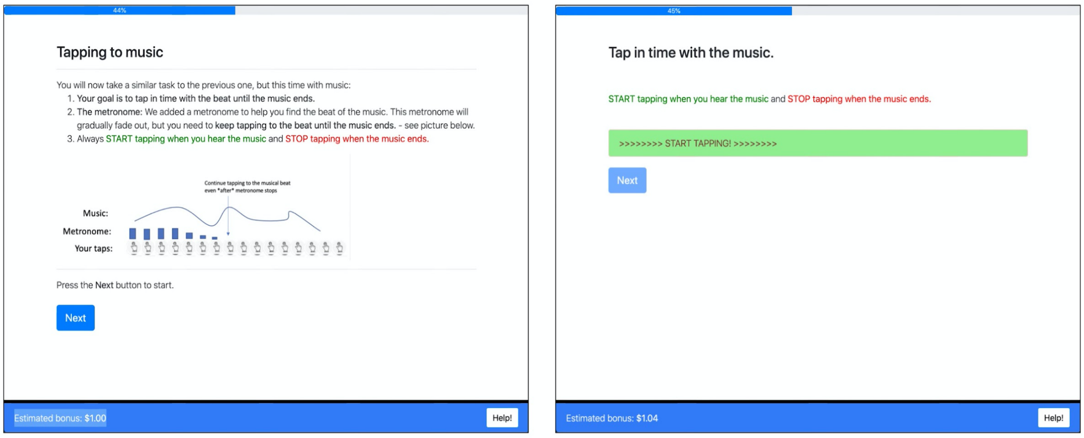

Figure S5. Instructions for the beat synchronization task

Failing Criteria

When measuring SMS in online experiments, it is crucial to determine whether participants are tapping in the required way (e.g., following the instructions) and whether any technical constraints may preclude the recording of their signal, such as cases with poor internet connection, malfunctioning hardware, or strong noise-cancellation technologies. To identify and exclude these cases in the online experiments reported in this paper (Experiment 3), we used two-step failing criteria. First, since REPP cannot work efficiently unless it detects all marker sounds with high precision, we failed all trials in which we could not detect all marker sounds included in the stimulus preparation step, or where the markers were displaced relative to each other for more than 15 ms. Second, we failed all trials where the percentage of detected taps (i.e., the number of detected tapping onsets out of the total number of stimulus onsets) was less than 50% or more than 200%. This measure is useful to deter participants from not responding at all or from tapping at an extremely fast rate, irrespective of the audio stimuli. Importantly, none of these criteria exclude trials based on actual tapping performance, but only based on whether the signal can be correctly recorded and processed by REPP and whether participants produced a minimally/maximally acceptable number of tapping responses.

In Experiment 3 (online), the failing criteria was used in the practice phase to exclude participants who did not provide at least two valid tapping trials. We also used the failing criteria in the main tapping tasks to fail individual tapping trials. Moreover, as a data cleaning step, we removed from the analysis all tapping trials where the markers were displaced relative to each other for more than 5 ms, ensuring that we only included cases with nearly optimal latency and jitter.

Custom Markers

REPP relies on custom markers located at the beginning and end of each stimulus to unambiguously identify the position of the tapping and stimulus onsets in the audio recording. We extensively piloted the parameters to generate and extract the marker onsets and found the following to work most efficiently across computer models and brands.

Generation procedure. The marker sound was created with a combination of a filtered white noise (50%) and pure tones (50%), namely:

Eq. S1

$$marker\_sound(t) = 0.5*G_{marker\_range}(t) + 0.5*F_{\mathrm{mean}(marker\_range)}(t),$$

Where, *marker_range=(marker_range(1), marker_range(2))* represents the frequency range of the resulting sound *marker_sound(t)*; $G_{marker\_range}(t)$ refers to white Gaussian noise that was bandpass filtered with a filter with cutoff frequencies of *marker_range(1)* and *marker_range(2)*, and $F_{\mathrm{mean}(marker\_range)}(t)$ refers to the pure tone with a frequency of mean*(marker_range)* defined as the geometric mean of the two frequencies in *marker_range*, namely:

Eq. S2

$$\mathrm{mean}(marker\_range) = \mathrm{sqrt}(marker\_range(1)*marker\_range(2))$$

We applied a linear attack and release of 2 ms and a total duration of 15 ms providing a crisp onset. The frequency range for the markers (*marker_range*) was set between 200 and 340 Hz and the markers were max normalized and scaled by 90%.

We then positioned three marker sounds at the beginning of the stimulus and three at the end. The marker sounds were positioned with IOIs at 0, 280, and 230 ms. To create the final target stimulus, we then shift the corresponding list of stimulus onsets to take into account the added markers (see Figure S6). In particular, we shift the stimulus onsets by using a time window of 2 seconds between the three markers at the start and the beginning of the stimulus, and a time window of 3 seconds between the three last markers and the end of the stimulus.

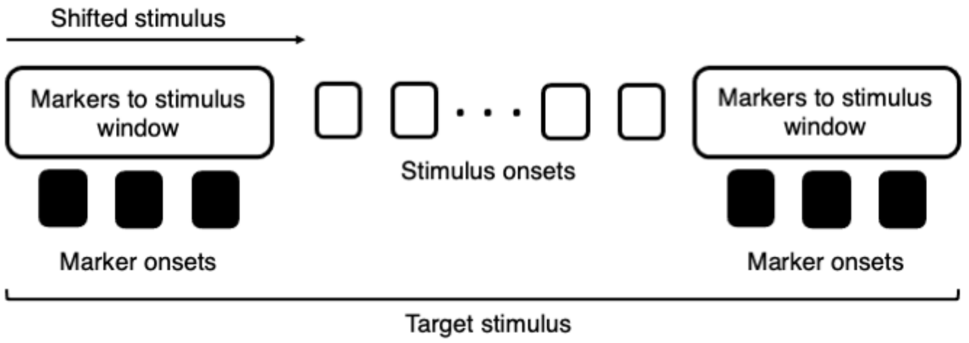

Figure S6. Diagram of the procedure to shift the stimulus onsets according to the marker onsets

The initial markers serve two main purposes: to indicate participants that the trial is about to start, and to align the stimulus onsets with the tapping onsets. In our technology, we only use the first marker onset to conduct the alignment procedure, but the code could be easily extended to use other alternatives (for example using all markers to provide increased accuracy). In particular, we calculate the time of each stimulus and tapping onset relative to the first marker. The markers at the end are used to notify participants that the trial is finished and calculate key metrics to assess the performance of our technology (see *Failing Criteria*). Specifically, (1) we assess the total number of detected markers, ensuring we only accept trials where the six markers are detected, and (2) calculate the markers' detection error, a metric that can be used to assess the timing accuracy of our technology, namely:

Eq. S3
max_marker_error = max*(*abs*(detected_marker_onsets - known_marker_onsets))*,

where *known_marker_onsets* refer to the known list of marker onsets used to generate the markers, and *detected_marker_onsets* refer to the list of detected marker onsets after the onset extraction and cleaning procedure. Thus, by only accepting trials where the markers' error is below a certain threshold (e.g., 5 or 10 ms), one can make sure the timing accuracy of the technology remains high in all trials.

Extraction procedure. The markers' extraction procedure consists of three steps: channel separation, cleaning heuristic, and onset extraction. In the first step, we extract the markers' channel from the raw recording by using a bandpass filter with cut-off frequencies set to the markers' range (200-340 Hz). To extract the envelope of the sound, we max normalize the markers' channel and perform a standard envelope extraction procedure (e.g., McDermott & Simoncelli, 2011).

A cleaning heuristic is then used to improve the resulting signal. Although this heuristic is not essential, it helps increase the robustness of the process with noisy

recording or with laptops using strong noise cancellation technologies. This heuristic relies on a test channel selected to be one octave below the markers' range (100-170 Hz), so it uses part of the spectrum that is not used by the marker channel but is close to it.

We first take the extracted markers and test channels and perform envelope extraction by computing the stimulus maximum in different bins, determined by a "cleaning bin window" parameter set to 100 ms. Second, we compute the ratio between the two signals in all bins. Namely:

Eq. S4
cleaning_ratio(t) = marker_channel(t) / test_channel(t)

Since this ratio could have extreme values (for example when *test_channel(t)* is close to zero), we compute a trimmed ratio defined as follows:

Eq. S5
trimmed_cleaning_ratio(t) = min*(*max*(cleaning_ratio(t), 1/max_cleaning_ratio), max_cleaning_ratio)*

Intuitively, this ratio is large exactly where there is more energy in the markers compared with the test range. We then use this ratio to boost the markers' signal exactly in bins where the ratio is high by point-multiplying the amplitude by the ratio in the bin. Note that we never boost more than the *max_cleaning_ratio* (set to 10) or less than 1/ *max_cleaning_ratio*. Effectively, we amplify the regions that are likely to contain the markers but up to a certain factor (determined by *max_cleaning_ratio)* so that outlier locations that are not markers will not be enhanced too much. The resulting signal has enhanced markers' amplitude, which helps combat signal attenuation as a result of noise cancelation and noisy backgrounds (for example overcoming loud abrupt transient background sounds that can compete with the markers in their loudness). Namely:

Eq. S6
*enhanced_markers_channel(t) = markers_channel(t)*trimmed_cleaning_ratio(t)*

Finally, we max normalize the enhanced markers' channel and apply a simple onset extraction algorithm to detect all samples exceeding a relative threshold set to 22.5%. We found this threshold to work robustly when testing participants in online experiments, but in other testing conditions it can be reduced.

Figure S7 shows four examples of the output of the markers' extraction procedure: The two examples on the top (rows ***a*** and ***b***) result in a successful extraction (i.e., all markers can be detected with high timing accuracy), whereas the two examples on the bottom (rows ***c*** and ***d***) show common cases where the markers' extraction procedure fails (i.e., some markers cannot be detected either due to a lack of signal or too much noise overlapping with the markers' channel).

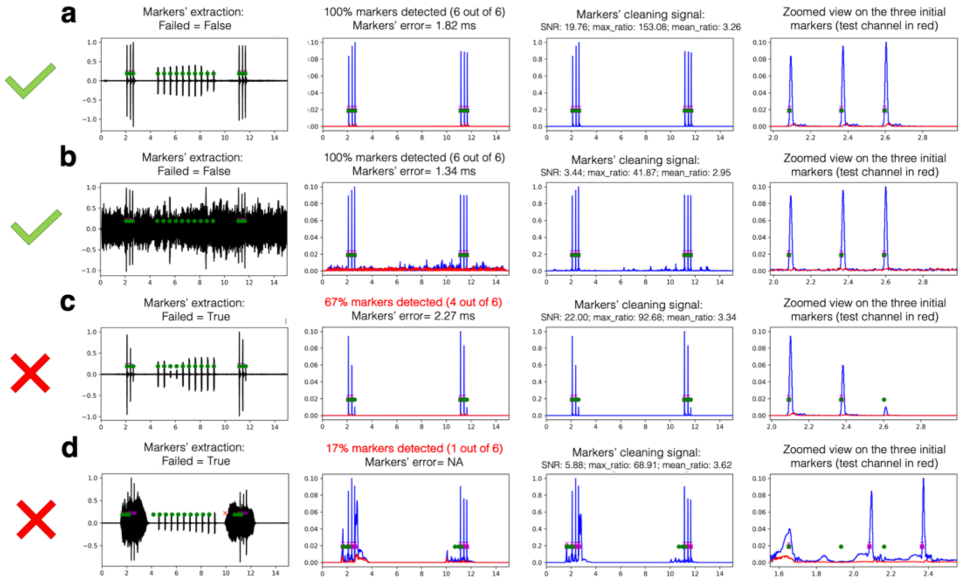

Figure S7. Examples of the markers' extraction procedure
a Example of a successful trial where all markers can be detected with high timing accuracy. In this example, the raw recording (left plot) is very clean and there is no floor noise. ***b*** Another example of a successful trial. This time, although the raw recording is noisy (left plot), the technology can still detect all makers with high timing accuracy. This is because the noise does not overlap with the frequency range of the markers' channel. ***c*** Example of a failed trial where the markers' extraction procedure cannot detect all markers. In particular, the amplitude of some marker onsets is too low to be detected by the onset extraction threshold (e.g., see the zoomed view in the right plot). ***d*** Another example of a failed trial where the extraction procedure cannot detect all markers. This time, there is external noise overlapping with the markers' range (i.e., 200-340 Hz), making it impossible to reliably detect the marker onsets (e.g., see the zoomed view in the right plot).

Trialing Alternative Measures of SMS

Throughout the paper, we used the SD of the tap-stimulus asynchrony to measure participants' tapping performance. Here, we repeat the main analyses reported in the two behavioral experiments (Experiment 2 and 3) using alternative measures of SMS. First, we repeated the main analyses using mean asynchrony instead of *SD* of asynchrony (see Figure S8), obtaining very similar results to the ones reported in Experiment 2 and 3 (see Figure 3 and 4, respectively).

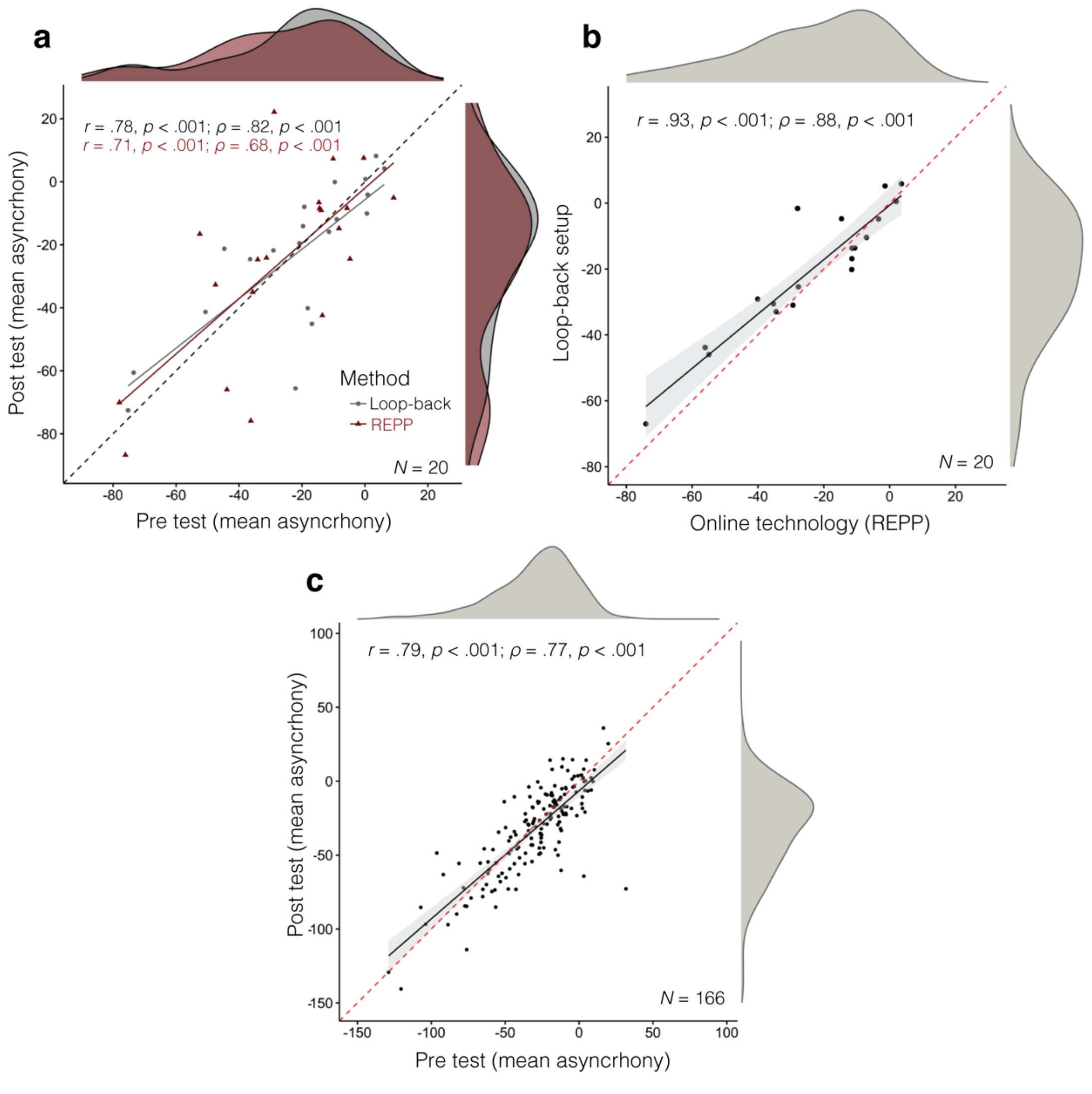

Figure S8. Replication of tapping accuracy analysis using mean asynchrony
a Experiment 2: test-retest reliability measured in the two methods. ***b*** Experiment 2: concurrent validity of REPP. ***c*** Experiment 3: test-retest reliability of REPP when measuring participants' tapping performance online.

Second, we repeated the main analyses using alternative measures of SMS, previously suggested in the literature. Namely:

1. Vector length was calculated to measure participants' synchronization using circular statistics (Fisher, 1993). The computation was performed on the unit circle, where 0 indicates unstable tapping with relative phases distributed uniformly (randomly) and 1 indicates perfect synchrony. For each response R_i we first identified the stimulus S_j that immediately precedes the response R_i. We next computed the phase associated with the response R_i using the following formula:

   Eq. S7

   $$\phi_i = \frac{R_i}{S_{j+1} - S_j}$$

   We then computed the average vector length on the complex plane:

   Eq. S8

   $$\underline{\phi} = |\Sigma_{i=1,\ldots,N} \exp\ (-2\pi i * \Phi_i)\ |/N$$

   The resulting value is typically high (>0.7) even for relatively poor tapping performance. We noticed that values below 0.5 indicated trials where participants did not perform the task as instructed (e.g., they did not try to synchronize to the stimulus cues or were confused about the starting cue, or). We therefore excluded trials with values lower than 0.5. Accordingly, a total of 15 trials in Experiment 1 (2.34 %) and 197 trials in Experiment 2 (6.35 %) were excluded from the analysis.

2. A leading model of SMS was proposed by Vorberg and Wing (Vorberg & Wing, 1996; Vorberg & Schulze, 2002) to estimate key sensorimotor and cognitive processes involved in synchronization while accounting for different sources of internal noise. The model is based on three hypothesized components: a *timekeeper noise*, reflecting the instability of representing time intervals, *motor noise* representing an independent component originated from the motor system, and *error correction, a* constant that allows adaptation to the metronome sequence. For isochronous tapping, the model can be written as:

   Eq. S9

   $$A_{t+1} = (1-\alpha)A_t + T_t + M_{t+1} - M_t + C,$$

   where, C is the constant metronome ISI, A_t is the asynchrony at time t, α is the phase correction constant, and var$(T_t) = \sigma_T^2$ and var$(M_t)=\sigma_M^2$ are the timekeeper and motor noise variances, respectively (these are parameters of

the model estimated from data). The model parameters can be estimated from the data using the bGLS methods described in Jacoby et al. (2015a). However, when tempo changes are introduced, one needs to use a more complicated model with an additional term estimating period correction. This model also requires more data to evaluate it reliably. Thus, we only used this model for the isochronous tapping task performed in Experiment 2 and 3.

3. *Lag-1 autocorrelation* of the asynchrony is a simpler measure of error correction. Here we estimate the correlation of the asynchrony at lag-1 (corr(A_t, A_{t-1})). Vorberg and Shultze (2002) suggest a link between this autocorrelation at lag-1 and the three hypothesized components of their model: timekeeper noise, motor noise, and phase correction (Vorberg and Shultze 2002; theorem 3.3).

4. *Lag-1 autocorrelation* of the inter tap interval (ITI), defined as corr(r_t, r_{t-1}), where r_t is the inter-response interval. It has been proposed that in synchronization this 1-lag should be negative, however it was empirically found to be dependent on the production modality (Ammirante et al., 2016) and inter-stimulus interval (Repp, 2011).

The results of all SMS measures considered in this study are provided in Table S1. From this, it is clear that the SD of the tap-stimulus asynchrony, mean asynchrony, and vector length are highly reliable and provide results with similar effect sizes across all analyses. In comparison, the more complex measures (i.e., timekeeper and motor noise, phase correction, and measures of lag-1 autocorrelation) are less reliable and more sensitive to features of the design and analysis, such as the type of tapping task, number of participants included in the analysis, and missing values. In particular, with a small number of participants (Experiment 2, N = 20), some of the more complex measures yield inconsistencies when comparing the results obtained in the in-lab method (the loop-back setup using MATLAB) and REPP. This can be partly explained by the influential effects of outliers when computing correlations with small sample sizes. In fact, the results of Experiment 3 using a larger sample of participants (N = 166) tend to be more consistent with those obtained using the MATLAB pipeline in Experiment 2. These inconsistencies may also arise from the slightly different thresholds used to include tapping onsets in each setup. For instance, the MATLAB algorithm is able to use a lower relative threshold to detect tapping onsets than REPP, as the signal's floor noise is lower due to the more sophisticated equipment (the loop-back setup). While robust measures of SMS are unaffected by these small differences (i.e., SD of asynchrony, mean asynchrony, and vector length), more complex measures are more sensitive to them. This is consistent with the finding that the reliability of the lag-1 autocorrelation of asynchrony is higher than the reliability of the lag-1 autocorrelation of ITI: the former only involves two onsets (the two consecutive asynchronies), whereas the later depends on more onsets (the three onsets involved in two consecutive ITIs).

Despite this, when considering the results in all analyses, the more complex measures of SMS generally provide reliable results, with the only exception found in two components from the Vorberg and Wing model: motor noise and error correction. Specifically, we found that timekeeper noise can be estimated more reliably than motor noise and phase correction. This is consistent with the idea that motor noise is very small and hard to estimate reliably (Jacoby et al., 2015b). However, it is apparent from these preliminary results that the current experiments were not powered enough to provide highly reliable estimates of these complex measures (see Jacoby et al. 2015a), both in terms of the number of trials per participant and tapping onsets per trial. We see great potential for future research using REPP for sophisticated individual differences modelling, as long as these studies use more robust individual difference tests with the right amount of data points per participant (e.g., Vishne et al. 2021).

Table S1. Alternative measures of SMS

Measure	Exp. 2 Concurrent Validity	Exp. 2 Reliability (Matlab)	Exp. 2 Reliability (REPP)	Exp. 3 Reliability (REPP - Online)
SD of asynchrony	$r = .94$<br>$\rho = .80$	$r = .89$<br>$\rho = .83$	$r = .87$<br>$\rho = .81$	$r = .80$<br>$\rho = .81$
Mean asynchrony	$r = .95$<br>$\rho = .90$	$r = .78$<br>$\rho = .83$	$r = .71$<br>$\rho = .68$	$r = .79$<br>$\rho = .77$
Vector length	$r = .89$<br>$\rho = .88$	$r = .65$<br>$\rho = .67$	$r = .46$<br>$\rho = .42$	$r = .67$<br>$\rho = .75$
Lag-1 cor. of asynchrony	$r = .69$<br>$\rho = .64$	$r = .65$<br>$\rho = .46$	$r = .67$<br>$\rho = .42$	$r = .66$<br>$\rho = .69$
Lag-1 cor. ITI	$r = .55$<br>$\rho = .34$	$r = .28$<br>$\rho = .19$	$r = .50$<br>$\rho = .47$	$r = .38$<br>$\rho = .40$
Timekeeper noise*	$r = .78$<br>$\rho = .74$	$r = .79$<br>$\rho = .72$	$r = .77$<br>$\rho = .77$	$r = .69$<br>$\rho = .69$
Motor noise*	$r = .15$<br>$\rho = .23$	$r = .56$<br>$\rho = .44$	$r = .45$<br>$\rho = .36$	$r = .43$<br>$\rho = .30$
Error correction*	$r = .40$<br>$\rho = .39$	$r = .71$<br>$\rho = .68$	$r = .33$<br>$\rho = .41$	$r = .50$<br>$\rho = .48$

Note. The analysis strategy to calculate concurrent validity and test-retest reliability was the same used in Experiment 2 and 3, respectively. For measuring test-retest reliability in Experiment 3, we only considered participants who provided at least one valid tapping trial for each stimulus in each tapping task and test-retest condition ($N = 166$).
*Indicates that the measure was from the Vorber and Wing model (Vorberg & Wing, 1996; Vorberg & Schulze, 2002), calculated as described in Jacoby et al. (2015a).

References

Ammirante, P., Patel, A. D., & Russo, F. A. (2016). Synchronizing to auditory and tactile metronomes: a test of the auditory-motor enhancement hypothesis. *Psychonomic bulletin & review, 23*(6), 1882-1890.

Clifford, S., & Jerit, J. (2014). Is there a cost to convenience? An experimental comparison of data quality in laboratory and online studies. *Journal of Experimental Political Science, 1*(2), 120.

Crump, M. J., McDonnell, J. V., & Gureckis, T. M. (2013). Evaluating Amazon's Mechanical Turk as a tool for experimental behavioral research. *PloS one*, *8*(3), e57410.

Fisher, N. I. (1993). Statistical analysis of circular data. Cambrdige, US: Cambridge University Press.

Harrison, P., Marjieh, R., Adolfi, F., van Rijn, P., Anglada-Tort, M., Tchernichovski, O., ... & Jacoby, N. (2020). Gibbs Sampling with People. *Advances in Neural Information Processing Systems*, *33*.

Jacoby, N., Tishby, N., Repp, B. H., Ahissar, M., & Keller, P. E. (2015a). Parameter estimation of linear sensorimotor synchronization models: phase correction, period correction, and ensemble synchronization. *Timing & Time Perception*, *3*(1-2), 52-87.

Jacoby, N., Keller, P. E., Repp, B. H., Ahissar, M., & Tishby, N. (2015b). Lower bound on the accuracy of parameter estimation methods for linear sensorimotor synchronization models. *Timing & Time Perception*, *3*(1-2), 32-51.

McDermott, J. H., & Simoncelli, E. P. (2011). Sound texture perception via statistics of the auditory periphery: evidence from sound synthesis. *Neuron*, *71*(5), 926-940.

McKinney, M. F., Moelants, D., Davies, M. E., & Klapuri, A. (2007). Evaluation of audio beat tracking and music tempo extraction algorithms. *Journal of New Music Research*, *36*(1), 1-16.

Repp, B. H. (2011). Comfortable synchronization of drawing movements with a metronome. *Human Movement Science, 30*, 18–39.

Vorberg, D., & Schulze, H. (2002). Linear phase-correction in synchronization: Predictions, param- eter estimation, and simulations. *Journal of Mathematical Psychology, 46*(1), 56–87.

Vorberg, D., & Wing, A. (1996). Modeling variability and dependence in timing. In H. Heuer & S.W. Keele (Eds.), *Handbook of perception and action* (Vol. 2, pp. 181–262). London, UK: Academic Press.

Vishne, G., Jacoby, N., Malinovitch, T., Epstein, T., Frenkel, O., & Ahissar, M. (2021). Slow update of internal representations impedes synchronization in autism. *Nature communications, 12*(1), 1-15.
